# Extracellular Vesicle-Encapsulated MicroRNA-375 from Bone Marrow-Derived Mesenchymal Stem Cells Inhibits Hepatocellular Carcinoma Progression through Regulating HOXB3-Mediated Wnt/*β*-Catenin Pathway

**DOI:** 10.1155/2022/9302496

**Published:** 2022-01-27

**Authors:** Zhaoxia Yu, Ju Liu, Qiqi Fan, Jun Yu, Xiaoting Ren, Xiaobin Wang

**Affiliations:** ^1^Department of Functional Examination, Qingdao Sixth People's Hospital, Qingdao 266003, Shangdong, China; ^2^Department of Ninth Liver Disease, Qingdao Sixth People's Hospital, Qingdao 266003, Shangdong, China

## Abstract

Nowadays, microRNA-375 (miR-375) has been implicated in many types of cancers, including hepatocellular carcinoma (HCC), and the functions of miRNAs encapsulated by extracellular vesicles (EV) in HCC progression have also been extensively investigated. In this research, we aimed to probe into the mechanism of EV-encapsulated miR-375 from bone marrow-derived mesenchymal stem cells (BM-MSCs) in HCC progression. At first, miR-375 expression in HCC tissues and cells was detected using RT-qPCR, and miR-375 was overexpressed to specify the effects of miR-375 on the malignant phenotype of HCC cells. miR-375 was downregulated in HCC, and overexpression of miR-375 suppressed HCC cell growth. Then, BM-MSCs and EV were isolated and identified, and, EV were cocultured with HCC cells for further functional assays. It was found that miR-375 encapsulated by EV could restrict the malignant phenotypes of HCC cells. Furthermore, the downstream genes and signaling cascades involved in HCC growth were investigated. HOXB3 was determined to be a downstream target of miR-375, and upregulation of miR-375 decreased Wnt1 and *β*-catenin protein expression. Furthermore, HOXB3 blocked the repressive effects of miR-375 on HCC cells and Wnt1 and *β*-catenin expression. This study highlights that miR-375 encapsulated by EV inhibits HCC development via modulating the HOXB3/Wnt/*β*-catenin axis.

## 1. Introduction

Hepatocellular carcinoma (HCC) is a common type of cancer with poor prognosis, and this disease is a major healthy burden around the world [1, 2]. The well-known risk factors for HCC include genetic metabolic diseases, toxins (e.g., aflatoxin), hepatitis B virus and hepatitis C virus hepatitis, and alcoholic and nonalcoholic fatty liver disease [3]. Generally, patients with HCC are prone to be diagnosed at an advanced stage due to the absence of pathognomonic symptoms, contributing to a poor prognosis [4]. The potential treatment regimens consist of resection, local ablative therapies, and liver transplantation, while these therapies are not applicable for patients at advanced stages [5] who exhibit therapeutic resistance and low response rate [6]. To ease these situations, effective biomarkers are needed for HCC in terms of early detection and prognosis.

Mesenchymal stem cells (MSCs) are a type pf extensively distributed cells showing potentials in self-renewing and multilineage differentiation [7]. Bone marrow-derived MSCs (BM-MSCs) are capable of repairing injured tissues, restraining the effector roles of immune cells, and converting them into regulatory cells [8]. Human BM-MSCs (h-BM-MSCs) are well-suited for clinical application because they are easily accessible and because autologous transplantation, which obviates immunologic incompatibilities, is possible [9]. Extracellular vesicles (EVs) represent an important mode of intercellular communication by serving as vehicles for transfer between cells of membrane and cytosolic proteins, lipids, and RNA [10]. EVs, classified into two main types: exosomes (exo) and microvesicles, are defined as lipid bilayer particles naturally released from cells into the extracellular space [11]. The importance of EV-incorporated noncoding RNAs (ncRNAs) to the HCC progression and their potent clinical applications in HCC have been summarized [12, 13]. MicroRNAs (miRNAs) are a kind of ncRNAs that mainly identify the complementary sequences in their target gene 3′-untranslated regions (UTRs), which results in mRNA degradation or translation suppression [14, 15]. Overexpression of miR-375 has been shown to promote the differentiation of human-induced pluripotent stem cells into hepatocyte-like cells [16]. The poor expression of miR-375 has been demonstrated in many types of cancers, which suppresses the cancer progression through targeting some important oncogenes [17, 18]. For instance, miR-375-3p can enhance the chemosensitivity to 5-fluorouracil in colorectal cancer [19]. Moreover, evidences have shown that miR-375 is downregulated in HCC, which could be a candidate prognostic biomarker of HCC [20–22]. Previously, HOXB3 has been verified to be directly bound by miR-375 in both pancreatic cancer [23] and breast cancer [24]. HOX genes regulate the cellular processes, and the HOXA13 deregulation is found to be involved in HCC [25]. Nevertheless, the target relationship between HOXB3 and miR-375 is scarcely investigated in HCC. Therefore, in this study, we aimed to probe into the regulatory mechanisms of EV-incorporated miR-375 on the occurrence of HCC with the involvement of HOXB3.

## 2. Materials and Methods

### 2.1. Ethical Approval

All patients signed the informed consents, and this study was permitted by the Ethics Committee of Qingdao Sixth People's Hospital. The collection and use of clinical specimens conformed to the *Declaration of Helsinki*.

### 2.2. Sample Collection

HCC tissues and their corresponding paracancerous tissues were harvested from 13 HCC patients who underwent tumor resection in Qingdao Sixth People's Hospital. Patient information is listed in Table 1. The samples were placed in liquid nitrogen immediately after collection and then stored at -80°C.

### 2.3. Cell Culture

Human HCC cell lines SNU-449 (American Type Culture Collection, Manassas, VA, USA) and Huh7 (China Center for Type Culture Collection, Wuhan, Hubei, China) were stored in Dulbecco's modified Eagle's medium (DMEM, Gibco, Invitrogen, Carlsbad, CA, USA), which contained 10% fetal bovine serum (FBS, Clark, Houston, TX, USA) and 1% antibiotics-antifungal solution (Life Technologies, Carlsbad, CA, USA).

### 2.4. Isolation, Culture, and Identification of h-BM-MSCs

Bone marrow cells were isolated from the femoral head of patients who had undergone hip replacement in Qingdao Sixth People's Hospital. The bone marrow was mixed with the culture medium (MesenPRO RS™ medium, Gibco, Grand Island, NY, USA, 12746-012) and separated by a h-BM-MSC isolation kit. The collected cells were spread in a tissue culture flask for 2-3 days, and the medium was renewed continuously to exhaust the medium. Seven days after the initial seeding, the monolayer cells were observed, and the expression of BM-MSC markers CD73 (ab202122), CD90 (ab23894), and CD105 (ab2529) and hematopoietic and endothelial cell markers CD34 (ab81289), CD11b (ab8878), CD19 (ab134114), CD45 (ab40763), and HLA-DR (ab92511) was detected by flow cytometry for the identification of h-BM-MSCs. All these markers were purchased from Abcam (Cambridge, MA, USA).

### 2.5. Isolation of EV

Before collecting the EV, the culture medium supplemented with FBS was centrifuged to remove excessive EV, and the BM-MSCs were cultured in the medium after centrifugation for 24-48 hours. The culture medium was collected, and the EV were harvested from 50 mL h-BM-MSC culture medium. The medium was placed on ice, centrifuged at 800 g for 10 minutes to pellet the cells, and then centrifuged at 12000 g for 20 minutes to remove cell debris. The EV were separated from the supernatant through centrifugation at 100,000 g for 2 hours, rinsed once in phosphate buffered saline (PBS), and then resuspended in 100 *μ*L PBS. The expression of markers CD9, CD63, and CD81 in isolated EV was examined by Western blot assay. EV structure was observed by transmission electron microscope (TEM), and the size distribution of EV was determined by nanoparticle tracking analysis (NTA). EV were quantified indirectly using a Pierce™ BCA Protein Quantification Kit (Thermo Fisher Scientific Inc., Waltham, MA, USA), and 50 *μ*g EV was used for each experiment.

### 2.6. EV Labeling

EV were stained with the green fluorescent dye PKH67 (MINI67-1KT, Sigma-Aldrich, St. Louis, MO, USA) and cocultured with recipient cells in 96-well plates, with PBS-treated cells serving as the controls. After 24 hours, the cells were washed with PBS to wash off the unbound EV. The cells were then fixed with 4% paraformaldehyde and stained for nuclei with Hoechst 33342. Subsequently, the uptake of EV by the recipient cells was observed by fluorescence microscopy.

### 2.7. Cell Grouping and Transfection

EV containing miR-375 mimic or negative control (NC) mimic were cocultured with SNU-449 and Huh7 cells, and then, SNU-449 and Huh7 cells were transfected with overexpressed (oe)-HOXB3 vector or NC, which was named the EV-NC mimic+oe-NC group, EV-miR-375 mimic+oe-NC group, EV-NC mimic+oe-HOXB3 group, or EV-miR-375 mimic+oe-HOXB3 group. The vectors used for cell transfection were designed and constructed by Shanghai Gene Pharmaceutical Co., Ltd. (Shanghai, China). The cocultured cells were seeded onto a 6-well plate overnight at 1 × 10^6^ cells/well. The vector (50 pmoL) was supplemented with a certain amount of serum-free diluent to a final volume of 25 *μ*L. The cell transfection was performed as per the kit instructions of Lipofectamine 2000 transfection reagent (Invitrogen, Carlsbad, CA, USA), and the transfected cells were harvested for subsequent experiments. For EV-miR-375 mimic-treated Huh7 cells, we further treated them with 20 nM sorafenib or dimethylsulfoxide (DMSO) for 24 h for following experiments.

### 2.8. Reverse Transcription Quantitative Polymerase Chain Reaction (RT-qPCR)

The total RNA was extracted from TRIzol, and the reverse transcription of a part of RNA was carried out using PrimeScript™ RT kit (Takara, Dalian, China). The complementary DNA (cDNA) after reverse transcription was used for subsequent expression detection of HOXB3 and glyceraldehyde phosphate dehydrogenase (GAPDH). Another part of RNA was taken, and reverse transcription was performed using the miRNA reverse transcription kit (Shanghai Haifang Biotechnology Co., Ltd., Shanghai, China). The cDNA after reverse transcription was adopted for subsequent expression detection of miR-375 and U6. RT-qPCR was performed with SYBR Premix Ex Taq II Kit (Takara). The primer information is listed in Table 2. GAPDH and U6 were selected as control genes of HOXB3 and miR-375, respectively. The 2^-∆∆Ct^ quantification method was used for data analysis.

### 2.9. Western Blot Assay

The total proteins were extracted from cold PBS-rinsed cells with radioimmunoprecipitation assay lysis buffer (P0013K, Beyotime, Shanghai, China). After adding protein loading buffer, the proteins were denatured in a boiling water bath and centrifuged to obtain the supernatant for subsequent detection. Next, the protein (20 *μ*g) was separated with sodium dodecyl sulfate-polyacrylamide gel electrophoresis and electroblotted onto polyvinylidene difluoride membranes. With 2-hour blocking, the membrane was probed with the primary antibodies against CD9 (MA5-31980), CD63 (MA5-32085), CD81 (MA5-32333), HOXB3 (PA5-103890) (all from Thermo Fisher), wnt1 (ab15251), and *β*-catenin (ab16051) (both from Abcam) overnight at 4°C, followed by 2-hour incubation with goat anti-rabbit antibody against IgG H&L (HRP) (ab6721, Abcam) at 37°C. Enhanced chemiluminescence (ECL) detection kit (Tanon, Shanghai, China) was utilized to observe protein bands. Finally, the protein was standardized with GAPDH (ab181602, Abcam) and quantified with an ECL chemiluminescence analyzer (Tanon). ImageJ program was implemented to analyze the gray value.

### 2.10. 5-Ethynyl-2′-deoxyuridine (EdU) Assay

For cell proliferation detection, HCC cells were incubated with the mixture of cell culture medium and EdU solution (1000 : 1) for 2 hours at room temperature, fixed with 40 g/L paraformaldehyde for 30 minutes, incubated with glycine solution for 8 minutes, and then washed with PBS containing 0.5% Triton X-100. Next, the cell culture plate was incubated at room temperature for 30 minutes with Apollo staining reaction solution and with Hoechst 33342 reaction solution for 20 minutes (both at room temperature devoid of light). The cell morphology was viewed under a fluorescence microscope. Green-stained cells were proliferating cells. When observing under the violet light of the excitation channel, the cells stained in blue were total cells. EdU-stained cells (proliferating cells) and Hoechst 33342-stained cells (total cell numbers) were counted from three random fields (cell proliferation rate = proliferating cells/total cell numbers × 100%).

### 2.11. Flow Cytometry

Cell apoptosis was evaluated by Annexin V-fluorescein isothiocyanate (FITC)/propidium iodide (PI) double staining. Briefly, the cells after 48-hour transfection were harvested, and the cell concentration was adjusted to 1 × 10^6^ cells/mL. Next, the cells were fixed with a prechilled 70% ethanol solution and incubated overnight at 4°C. Cell suspension (100 *μ*L, no less than 10^6^ cells/mL) was centrifuged, resuspended in 200 *μ*L binding buffer, and reacted with 10 *μ*L Annexin V-FITC and 5 *μ*L PI at room temperature without light exposure for 15 minutes. After the addition of 300 *μ*L binding buffer, the cells were loaded onto a flow cytometer (Attune NxT, Thermo Fisher Scientific) to detect cell apoptosis at an excitation wavelength of 488 nm.

### 2.12. Sphere Formation Assay

The cells were dissociated with the trypsin (Gibco, Carlsbad, CA, USA) containing no ethylenediaminetetraacetic acid (EDTA) and no phenol red. The cells were washed with serum-free medium DMEM/F12. A total of 1 × 10^3^ cells were placed in the low-adhesion six-well plates (Corning Glass Works, Corning, N.Y., USA) and cultured in the serum-free medium DMEM/F12 containing 20 ng/mL epidermal growth factor and 10 ng/mL basic fibroblast growth factor. All images were observed and photographed by an inverted microscope (Olympus Optical Co., Ltd., Tokyo, Japan). The numbers of spheres formed were counted.

### 2.13. Colony Formation Assay

The cells were dissociated and counted, and 1 × 10^3^ cells were seeded in each well of a 6-well plate with 2 mL culture medium. The culture medium was replaced every 4 days for 2 weeks. The medium was subsequently removed, and the cells were fixed in 4% paraformaldehyde and stained with 0.05% crystal violet. The colonies formed (more than 50 cells) were observed, imaged, and counted.

### 2.14. Transwell Assay

A Transwell chamber (pore size 8 *μ*m; Corning) coated with Matrigel (BD Biosciences, San Jose, CA, USA) was used to detect cell invasion, while the Transwell chamber without Matrigel was utilized to detect cell migration. The cells at 48 hours posttransfection were seeded in the apical chamber with serum-free medium, and medium containing 10% FBS was seeded in the basolateral chamber. After incubation for 24 hours, the noninvasive cells which were on the upper surface of the membrane were wiped off with a cotton swab, and the invaded cells on the lower surface were fixed with methanol and stained with crystal violet solution. Finally, the number of migrated cells and invaded cells of each layer of membrane in 5 random fields of view were counted under a microscope.

### 2.15. Animal Experiments

Animal experiments were granted by Animal Care and Use Committee of Qingdao Sixth People's Hospital in compliance with institutional guidelines for the care and use of animals. This experiment used 12 male BALB/c nude mice (4 weeks old, about 15 grams, Beijing Huafukang Experimental Animal Co., Ltd., Beijing, China). Huh-7 Cells in the logarithmic growth phase were dissociated with trypsin (Gibco) without phenol red and EDTA and resuspended in PBS containing 50% Matrigel (BD Biosciences). Cell suspension (2 × 10^6^ cells/mL) was injected subcutaneously into the dorsal side of the BALB/C nude mice. The mice were euthanized 4 weeks later. Histopathological analysis was performed on tumor samples from mice.

### 2.16. Immunohistochemistry

The tissues were fixed, dehydrated, and paraffin-embedded in the 10% formalin to make tissue sections of about 5 *μ*m. The sections were dewaxed twice with xylene (15 minutes each time), followed by incubation in 3% H_2_O_2_ (Sigma-Aldrich) at 37°C for 30 minutes and boiling in 0.01 M citric acid buffer at 95°C for 20 minutes. After being blocked with serum working solution at 37°C for 10 minutes, the sections were incubated with diluted primary antibody to KI67 (1 : 1000, ab6721, Abcam) at 37°C for 2 h and with HRP-conjugated secondary immunoglobulin G (IgG) antibody and counterstained with hematoxylin (Meilunbio, China) at room temperature for 4 minutes. The sections were observed using a microscope (Olympus).

### 2.17. Dual-Luciferase Reporter Gene Assay

A dual-luciferase reporter gene assay was performed in order to detect the binding site between miR-375 and HOXB3. Constitutive firefly luciferase gene expression was provided by the psiCHECK-2 vector, which was utilized as an internal control. The cells (1 × 10^5^ cells/each well) were seeded onto a 24-well plate and incubated in 1 mL DMEM containing 15% FBS under the conditions of 5% CO_2_ at 37°C for 24 hours. HOXB3 wild-type (HOXB3-wt) and HOXB3-mutant (HOXB3-mt) were cotransfected into 293T cells with NC mimic or miR-375 mimic. After 48 hours of transfection, the cells were collected from each well, gently stirred for 15 minutes at room temperature, and lysed in 100 *μ*L of passive lysis buffer. The lysate containing 50 *μ*L luciferase assay reagent II and 50 *μ*L terminator was put into a luminometer tube. The efficacy or activity of luciferase was detected by the GloMax fluorescence reader (GloMax Multi Detection System).

### 2.18. Statistical Analysis

All statistical analyses were performed using SPSS software (version 18.0; SPSS Corporation, Chicago, IL, USA). All data were expressed as mean ± standard deviation of at least three independent experiments. Data analysis between the two groups was performed by *t*-test, and data analysis among multiple groups was performed by two-way analysis of variance (ANOVA). *p* < 0.05 referred to statistical significance.

## 3. Results

### 3.1. miR-375 Is Downregulated in HCC Tissues and Cells, and miR-375 Suppresses the Occurrence of HCC

**Figure 1:**
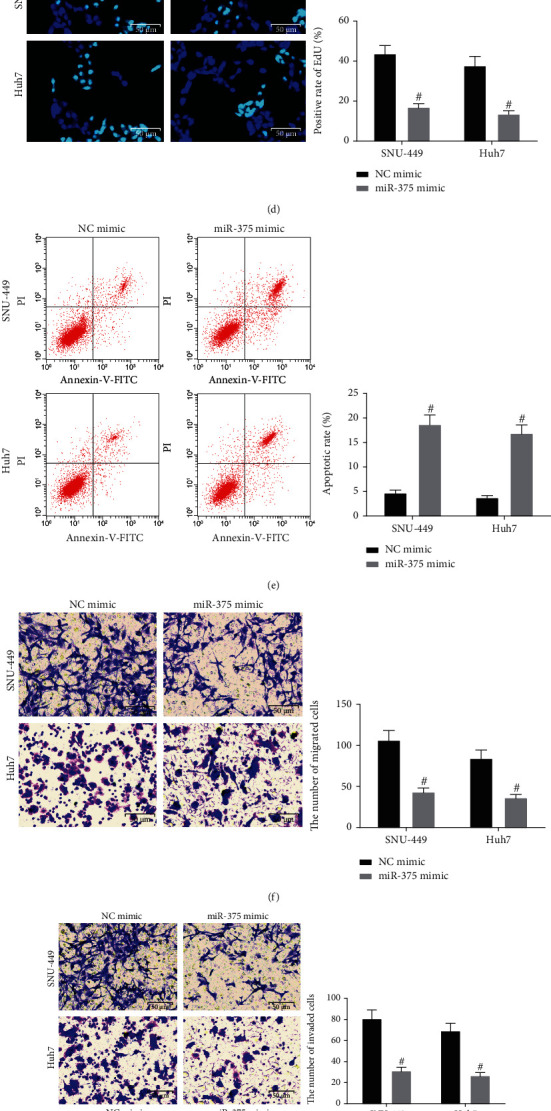
miR-375 expression is reduced in HCC, and overexpression of miR-375 retards the HCC occurrence. (a) miR-375 expression in HCC tissues and their corresponding paracancerous tissues of 13 patients with HCC was detected by RT-qPCR. (b) The correlation between miR-375 and patients' survival in the TCGA-LIHC database analyzed using Kaplan-Meier analysis. (c) miR-375 expression in the cells after transfection using RT-qPCR. (d) Cell proliferation in HCC cells using EdU assay; (e). Cell apoptosis rate in HCC cells using flow cytometry; (f). Cell migration ability in HCC cells using Transwell assay. (g) Cell invasion ability in HCC cells using Transwell assay. (h) Stem cell properties of HCC cells using sphere formation assay. (i) The number of colonies formed by HCC cells using colony formation assay. ^∗^*p* < 0.05 vs. paracancerous tissues; ^#^*p* < 0.05 vs. the NC mimic group. All data were expressed as mean ± standard deviation of at least three independent experiments. Data analysis between the two groups was performed by paired *t*-test, and data analysis among multiple groups was performed by two-way ANOVA.

Firstly, miR-375 expression in HCC tissues and their corresponding paracancerous tissues of 13 patients with HCC was detected by RT-qPCR, and we observed that miR-375 was significantly downregulated in HCC tissues of 13 patients (Figure 1(a)). We further analyzed the correlation between miR-375 expression and survival of patients in The Cancer Genome Atlas- (TCGA-) liver hepatocellular carcinoma (LIHC) by Kaplan-Meier analysis, and we found that LIHC patients with high miR-375 expression had higher survival rate (Figure 1(b)). The correlation between miR-375 expression and the TNM stage and tumor size was analyzed in clinically collected HCC patients. The patients with poor miR-375 expression had advanced tumor stage (Table 3).

In order to clarify the regulatory effect of miR-375 on HCC cells, we transfected SNU-449 and Huh7 with NC mimic or miR-375 mimic. RT-qPCR was conducted to detect miR-375 expression in the cells of each group, and the results indicated that miR-375 expression was elevated in cells upon treatment of miR-375 mimic (Figure 1(c)). EdU assay and flow cytometry were implemented to determine HCC cell viability and apoptosis, and the findings suggested that miR-375 mimic transfection in HCC cells resulted in a reduction in the proliferation rate and an elevation in the apoptosis rate (Figures 1(d) and 1(e)). Moreover, Transwell assay results exhibited that ectopic expression of miR-375 repressed the invasive and migratory potentials of HCC cells (Figures 1(f) and 1(g)). We further analyzed the effect of miR-375 on the stem cell properties of HCC cells using sphere formation assay as well as colony formation assay. The number of spheres formed and the number of colonies formed by SNU-449 and Huh7 cells overexpressing miR-375 had a significant reduction (Figures 1(h) and 1(i)). The above results imply that miR-375 expression is reduced in HCC, and overexpression of miR-375 inhibits the HCC occurrence.

### 3.2. BM-MSCs Are Successfully Extracted and Transfected with miR-375 Mimic for EV Isolation

**Figure 2:**
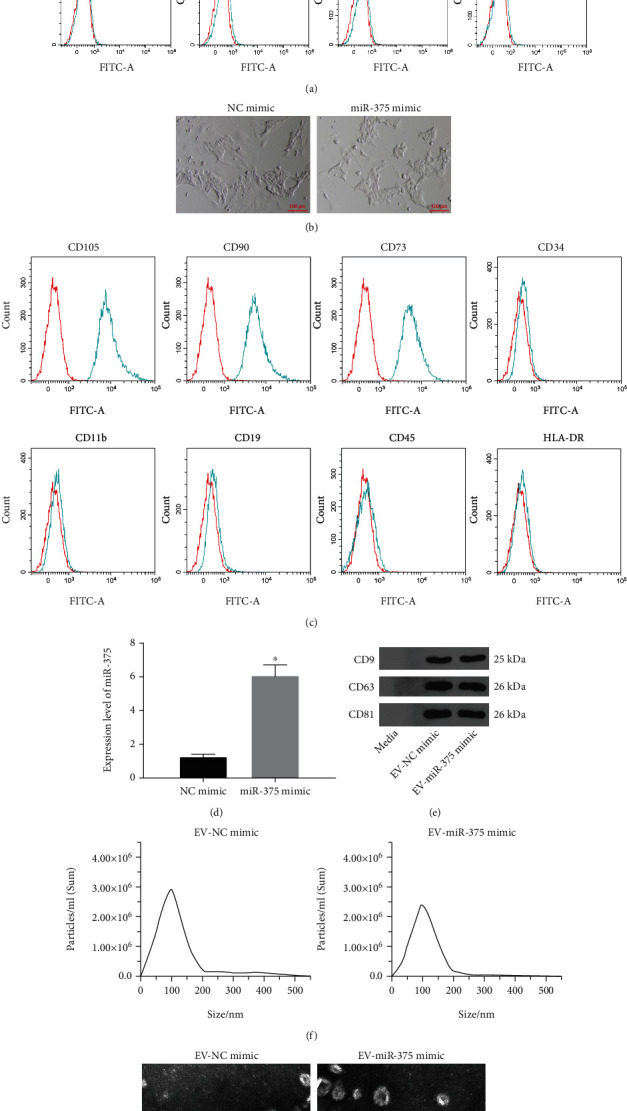
EV from miR-375 overexpression-modified BM-MSCs are successfully extracted. (a) Expressions of BM-MSC markers CD73, CD90, and CD105 and hematopoietic and endothelial cell markers CD34, CD11b, CD19, CD45, and HLA-DR were measured by flow cytometry. (b). Morphological observation of BM-MSCs. (c) Expression of BM-MSC markers CD73, CD90, and CD105 and hematopoietic and endothelial cell markers CD34, CD11b, CD19, CD45, and HLA-DR in BM-MSCs transfected with NC mimic and miR-375 mimic were measured by flow cytometry. (d) miR-375 expression in BM-MSCs after transfection using RT-qPCR. (e) Expression of EV markers CD9, CD63, and CD81 was determined by Western blot assay. (f) The size distribution of EV analyzed using NTA. (g) TEM observation of EV structure. ^∗^*p* < 0.05 vs. the NC mimic group. All data were expressed as mean ± standard deviation of at least three independent experiments. Data analysis between the two groups was performed by unpaired *t*-test.

We isolated BM-MSCs and determined the expression of BM-MSC markers (CD73, CD90, and CD105), hematopoietic and endothelial cell markers (CD34, CD11b, CD19, and CD45), and HLA-DR by flow cytometry. It was found that the expression of CD73, CD90, and CD105 was positive, and the expression of CD34, CD11b, CD19, CD45, and HLA-DR was negative (Figure 2(a)), suggesting that BM-MSCs were successfully isolated. Subsequently, the BM-MSCs were transfected with NC mimic or miR-375 mimic, and the cell morphology did not change after transfection (Figure 2(b)). We further detected the abovementioned markers by flow cytometry, and the results were consistent with the above, indicating that the morphology and function of BM-MSCs did not change after transfection (Figure 2(c)). Afterwards, miR-375 expression in BM-MSCs was detected by RT-qPCR, and the results showed that miR-375 was successfully overexpressed in BM-MSCs after transfection (Figure 2(d)). Next, we separated EV from BM-MSCs after transfection (named EV-NC mimic and EV-miR-375 mimic, respectively). Expression of CD9, CD63, and CD81 was determined in these extracted EV using Western blot assay, and the results revealed that expression of markers was positive in the NC mimic group and miR-375 mimic group (Figure 2(e)). NTA was implemented to detect the size distribution of EV. No change was observed in the size distribution of EV between the NC mimic group and the miR-375 mimic group (Figure 2(f)). The typical structure of lipid bilayer-delimited particles was observed in both EV by TEM (Figure 2(g)).

### 3.3. BM-MSCs Deliver miR-375 to HCC Cells by Secreting EV

**Figure 3:**
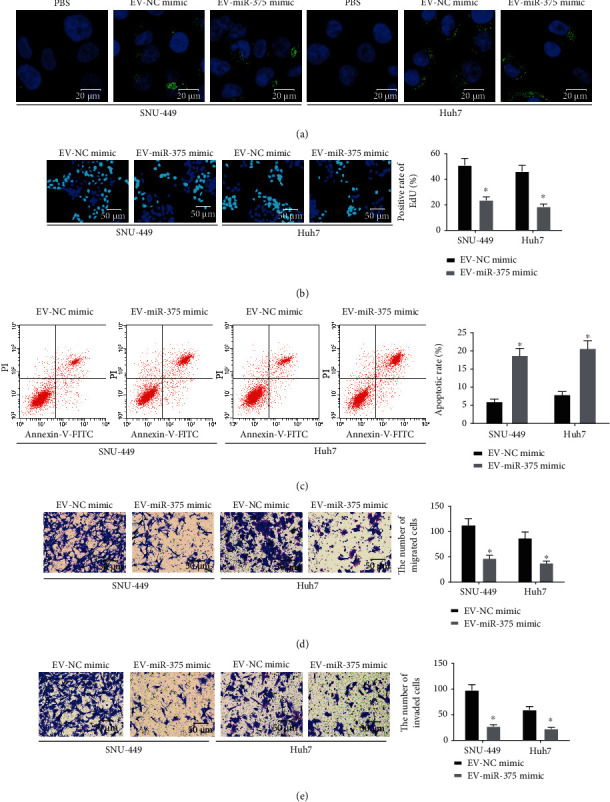
BM-MSCs secrete EV to deliver miR-375 to HCC cells. (a) Representative images of HCC cells under a fluorescence microscope to detect EV uptake by HCC cells. (b) Cell proliferation in HCC cells using EdU assay. (c) Cell apoptosis rate in HCC cells using flow cytometry. (d) Cell migration ability in HCC cells using Transwell assay. (e) Cell invasion ability in HCC cells using Transwell assay. ^∗^*p* < 0.05 vs. the EV-NC mimic group. All data were expressed as mean ± standard deviation of at least three independent experiments. Data analysis among multiple groups was performed by two-way ANOVA, followed by Tukey's multiple comparison test.

We cocultured the EV-NC mimic and EV-miR-375 mimic with HCC cell lines SNU-449 and Huh7, respectively. We detected the successful uptake of EV by the recipient cells after 24 h of EV treatment under a fluorescence microscope (Figure 3(a)). EdU assay, flow cytometry, and Transwell assay were conducted to determine HCC cell proliferation, apoptosis, migration, and invasion, and the results demonstrated that EV-miR-375 mimic led to a reduction in the proliferation rate, an elevation in the apoptosis rate, and a decrease in the number of migratory and invasive cells in HCC cells (Figures 3(b)–3(e)). The above results indicate that BM-MSCs secrete EV to deliver miR-375 to HCC cells, thereby inhibiting the HCC occurrence.

To further clarify the clinical potential of miR-375 incorporated by EV, we used EV in combination with sorafenib, a targeted drug for clinical treatment of HCC, to treat Huh7 cells. It was observed that the killing effect of EV-incorporated miR-375 on Huh-7 cells was significantly enhanced with the combination of sorafenib (Figure S1A-B).

### 3.4. EV-Derived miR-375 Inhibits the Growth of HCC Cells In Vivo

**Figure 4:**
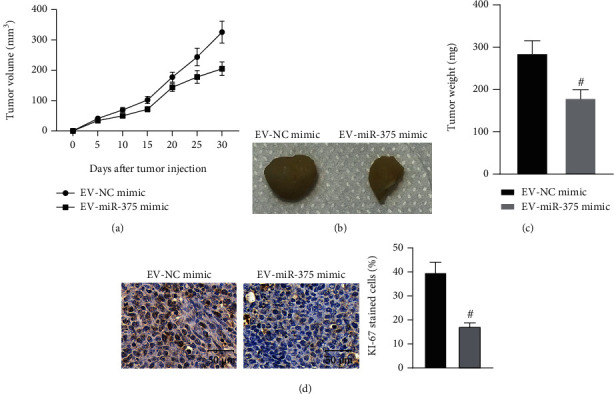
EV-derived miR-375 suppresses the growth of HCC cells *in vivo*. (a) Growth rate of xenograft tumors. (b). Representative images of xenograft tumors. (c) Tumor weight. (d) Intensity of KI67 staining in xenograft tumors using immunohistochemical staining. ^#^*p* < 0.05 vs. the EV-NC mimic group. All data were expressed as mean ± standard deviation (*n* = 6). Data analysis between the two groups was performed by unpaired *t*-test.

To further clarify the effect of EV-incorporated miR-375 on the growth of HCC cells *in vivo*, we injected Huh-7 cells into nude mice, followed by injection of EV-NC mimic or EV-miR-375 mimic every three days. We observed that the growth rate of EV-miR-375 mimic-treated xenograft tumors was significantly inhibited (Figures 4(a)–4(c)). We also found a significant reduction in the number of KI67-positive cells in EV-miR-375 mimic-treated xenograft tumors (Figure 4(d)). The above results show that EV-derived miR-375 repressed the growth of HCC cells *in vivo*.

### 3.5. EV-Derived miR-375 Targets HOXB3 in HCC Cells

**Figure 5:**
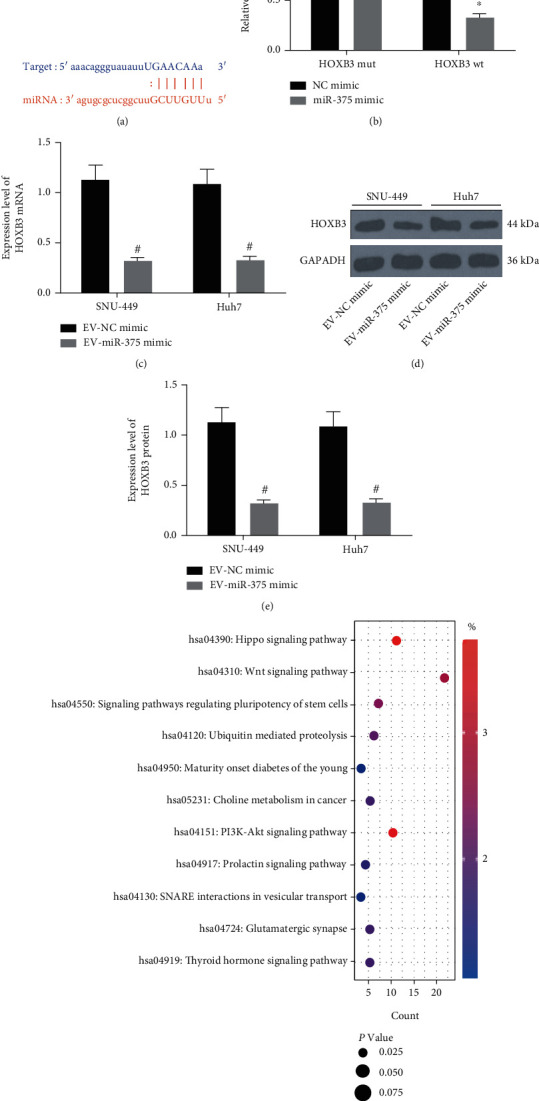
HOXB3 is found to be a downstream target of miR-375. (a) Binding sites between miR-375 and HOXB3 predicted through an online website. (b) The target relationship between miR-375 and HOXB3 was confirmed by dual-luciferase reporter gene assay. (c) HOXB3 mRNA expression in SNU-449 and Huh7 cells using RT-qPCR. (d, e) HOXB3 protein expression in SNU-449 and Huh7 cells using Western blot assay. (f) Enrichment analysis of miR-375 targeted genes using KEGG enrichment analysis. ^∗^*p* < 0.05 vs. the NC mimic group; ^#^*p* < 0.05 vs. the EV-NC mimic group. All data were expressed as mean ± standard deviation of at least three independent experiments. Data analysis among multiple groups was performed by two-way ANOVA, followed by Tukey's multiple comparison test.

In order to explore the regulatory mechanism of miR-375 in HCC cells, we first searched the downstream targets of miR-375 through the online website StarBase (http://starbase.sysu.edu.cn/), and a binding site was found between miR-375 and HOXB3 (Figure 5(a)). The dual-luciferase reporter gene assay further confirmed that miR-375 could target HOXB3 (Figure 5(b)). In addition, we performed RT-qPCR (Figure 5(c)) and Western blot assay (Figures 5(d) and 5(e)) to detect HOXB3 expression in SNU-449 and Huh7 cells cocultured with EV-NC mimic and EV-miR-375 mimic. Results suggested that reduced HOXB3 expression was observed in SNU-449 and Huh7 cells treated with EV-miR-375 mimic. We analyzed the correlation between HOXB3 and TNM stage and tumor size of clinically collected HCC patients, and patients with high HOXB3 expression had advanced tumor stage (Table 4). Moreover, the target genes of miR-375 were analyzed using Kyoto Encyclopedia of Genes and Genomes (KEGG) enrichment analysis, and we found that the genes targeted by miR-375 were mainly enriched in the Wnt signaling pathway (Figure 5(f)).

### 3.6. EV-Derived miR-375 Hinders the Development of HCC by Regulating the HOXB3/Wnt/*β*-Catenin Axis

**Figure 6:**
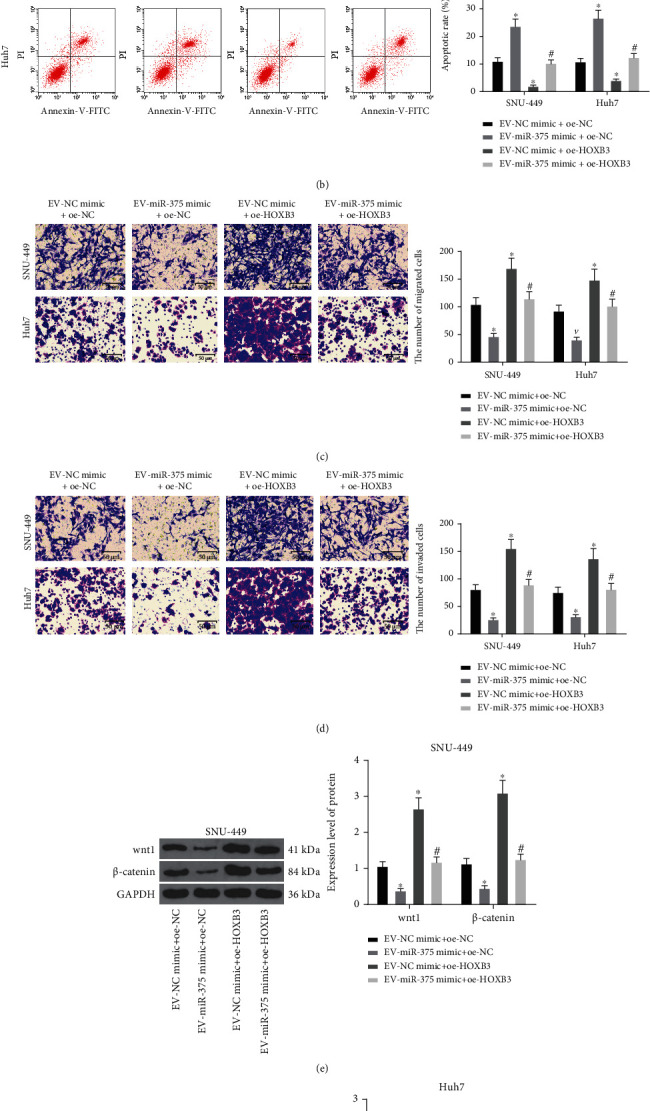
EV-derived miR-375 modulates HCC cell proliferation, apoptosis, migration, and invasion through regulating the HOXB3/Wnt/*β*-catenin axis. (a) Cell proliferation in HCC cells using EdU assay. (b) Cell apoptosis rate in HCC cells using flow cytometry. (c) Cell migration ability in HCC cells using Transwell assay. (d) Cell invasion ability in HCC cells using Transwell assay. (e) HOXB3 protein expression in SNU-449 cells using Western blot assay. (f) HOXB3 protein expression in Huh7 cells using Western blot assay. ^∗^*p* < 0.05 vs. the EV-NC mimic+oe-NC group; ^#^*p* < 0.05 vs. the EV-miR-375 mimic+oe-NC group. All data were expressed as mean ± standard deviation of at least three independent experiments. Data analysis among multiple groups was performed by two-way ANOVA, followed by Tukey's multiple comparison test.

SNU-449 and Huh7 cells after coculture were transfected with oe-HOXB3 or oe-NC. HCC cell malignant phenotypes were determined by EdU assay, flow cytometry, and Transwell assay, and the results revealed that relative to the EV-NC mimic+oe-NC treatment, the EV-miR-375 mimic+oe-NC treatment reduced proliferation rate, increased apoptosis rate, and decreased number of migratory and invasive cells in HCC cells. By contrast, EV-NC mimic+oe-HOXB3 delivery contributed to the opposite trends. Additionally, elevated proliferation rate, reduced apoptosis rate, and increased number of migratory and invasive cells were found in HCC cells treated with the EV-miR-375 mimic+oe-HOXB3 group versus those treated with EV-miR-375 mimic+oe-NC (Figures 6(a)–6(d)). Furthermore, we determined the expression of Wnt/*β*-catenin signaling pathway-related proteins in each group of cells by Western blot assay (Figures 6(e) and 6(f)). The findings demonstrated that in contrast to the EV-NC mimic+oe-NC administration, Wnt1 and *β*-catenin expression was found to be decreased following the EV-miR-375 mimic+oe-NC administration, which was increased following the EV-NC mimic+oe-HOXB3 administration. Additionally, elevated expression of Wnt1 and *β*-catenin was found in HCC cells treated with the EV-miR-375 mimic+oe-HOXB3 group versus those treated with EV-miR-375 mimic+oe-NC. In summary, it is suggested that EV-derived miR-375 can affect HCC cell malignant phenotypes via modulating the HOXB3/Wnt/*β*-catenin axis.

## 4. Discussion

miRNAs are known to exert fundamental functions in the modulation of multiple oncogenes or tumor suppressors, thereby being regarded as potent diagnostic biomarkers for cancer detection [26]. Thus, it is urgent to exploring novel targets of early diagnosis, timely monitoring, and effective treatment for HCC. In this current research, miR-375 was found to be downregulated in HCC and has a role in reverting the malignant phenotype of HCC cells both *in vitro* and *in vivo*. Moreover, tumor-suppressive effects of miR-375 were elicited through EV secretion from BM-MSCs. Mechanistically, miR-375 targeted HOXB3 to modulate the Wnt/*β*-catenin pathway. Our results, therefore, identified a new mechanism by which BM-MSCs-derived EV play a tumor suppressor in HCC via a miR-375-dependent manner.

In the past decades, evidence has focused on elucidating genes and proteins underlying HCC development [27]. Functional studies further substantiated their significant roles in hepatocarcinogenesis because of their abilities of controlling cell survival, proliferation, and invasive properties [28, 29]. As to HCC, miR-375 has been implied to be poorly expressed in HCC tissues, and miR-375 could suppress HCC cell proliferation and invasion *in vitro* [21]. In addition, miR-375 is decreased in HCC cell lines and tissues, and miR-375 could induce G1 arrest and apoptosis, while decreasing HCC cell proliferation, clonogenicity, migration, and invasion [20]. In our study, we have detected miR-375 downregulation in HCC tissues and cells, and overexpression of miR-375 inhibited the HCC occurrence. For clarifying the regulatory effect of miR-375 on HCC cells, NC mimic and miR-375 mimic were transfected into BM-MSCs. Subsequently, we cocultured the BM-MSCs-isolated EV with HCC cells. The results indicated that EV-miR-375 mimic could suppress the progression in HCC cells, implying that BM-MSCs secrete EV to deliver miR-375 to HCC cells, thereby inhibiting the HCC occurrence. It is reported that exosome-carried miR-375 restricts cell progression and dissemination in colon cancer and small cell lung cancer [30, 31]. Intriguingly, miR-375 has been reported to sensitize HCC cells to sorafenib by blocking sorafenib-induced autophagy [32]. Our functional assays also substantiated that sorafenib further enhanced the antitumor effects of EV-miR-375 mimic on HCC cells relative to DMSO, implying the possible clinic application of BM-MSC-derived EV. However, the function of EV-derived miR-375 in HCC needs further validation.

Targeting miRNA for therapeutic purposes has been revealed to simultaneously influence many genes and signaling cascades during modulating HCC growth [33]. miR-375, as one of these miRNAs, exhibits tumor suppressor activity in HCC by targeting epidermal growth factor receptor-2 and astrocyte elevated gene-1, respectively [20, 34]. We then took further steps to find the downstream target genes of miR-375 in HCC cells using the methods including online website prediction and luciferase activity assay. The findings of our study demonstrated that HOXB3 was negatively modulated by miR-375 in HCC. As for the target relationship between miR-375 and HOXB3, it has been elicited in the development of pancreatic cancer and breast cancer [23, 24], which is in line with our finding. In addition, our study also found that an elevation of HOXB3 could block the suppressive effect of EV-miR-375 on HCC cell malignant phenotypes. Despite the absence of articles on HOXB3 and HCC progression, many studies have correlated HOXB3 with other types of cancers. For instance, restoration of HOXB3 has been revealed to induce the ability of breast cancer cell migration and invasion [24]. Moreover, overexpression of HOXB3 partially reverses miR-375-induced suppression of proliferation and colony formation, suggesting that HOXB3 is of great importance in miR-375-induced antileukemia activity [35].

Although the Wnt/*β*-catenin pathway is often activated in HCC, the mechanism of its activation remains to be unearthed [36]. In this current research, we observed that miR-375 downregulated the expression of Wnt1 and *β*-catenin proteins. In accordance with our findings, transfection of miR-375 inhibitor could induce the activation of the Wnt/*β*-catenin pathway in gastric cancer cells [37]. The suppressive effect of exosomal miR-375-3p on the development of bladder cancer was achieved through the Wnt/*β*-catenin pathway inhibition [38]. Moreover, another article has suggested that circ-PRKDC deficiency leads to a reduction in *β*-catenin expression in bladder cancer cells, while miR-375 deletion rescued the effects [39]. All these data confirmed our findings to some extent.

## 5. Conclusion

**Figure 7:**
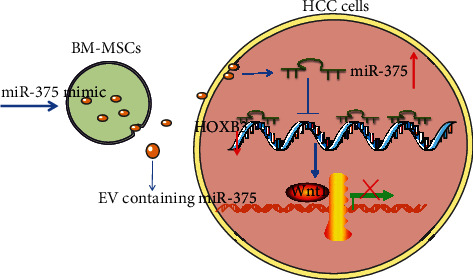
Schematic diagram depicting the molecular mechanism of EV-incorporated miR-375 in HCC cell proliferation, migration, and invasion. BM-MSC-derived EV inhibits HCC cell proliferation, migration, and invasion and promotes apoptosis by delivering miR-375 to HCC cells. miR-375 targets and inhibits the expression of HOXB3, thus impairing the downstream Wnt/*β*-catenin pathway.

In conclusion, this study provides evidence that miR-375 released from BM-MSC-derived EV targets HOXB3 to inhibit its expression and then suppresses the downstream Wnt/*β*-catenin pathway activation, thereby inhibiting the malignant phenotypes of HCC cells (Figure 7). Taken together, this study suggests that EV-derived miR-375 may serve as a novel therapeutic target for HCC patients. A potential pitfall of this work may be the way the dose of EV was quantified, and light scattering technologies or flow cytometry should be used in our further studies to measure the particle number.

## Figures and Tables

**Table 1:** Clinical baseline information for patients.

Pathological characteristics	Number of patients (*n*)
Age (year)	
<50	9
≥50	4
Gender	
Male	7
Female	6
Tumor size (cm)	
<3	3
≥3	10
Tumor stage (T)	
I-II	8
III-IV	5

**Table 2:** Primer sequence.

Name	Sequence (5′-3′)
miR-375	F: AGCCGTCAAGAGCAATAACGAA
	R: GTGCAGGGTCCGAGGT
HOXB3	F: ACCACCTTTCCCATCACCC
	R: GGCGCTTCTTGGATTCTACC
U6	F: CTCGCTTCGGCAGCACA
	R: AACGCTTCACGAATTTGCGT
GAPDH	F: CACCAGGGCTGCTTTTAACTC
	R: GAAGATGGTGATGGGATTTC

Note: F: forward; R: reverse; miR: microRNA; HOXB3: homeobox protein Hox-B3; GAPDH: glyceraldehyde phosphate dehydrogenase.

**Table 3:** The correlations of the miR-375 level with HCC clinical features.

Clinical features	*n*	High miR-375 (*n* = 7)	Low miR-375 (*n* = 6)	*p* value
Age (years)				
<50	9	5	4	>0.9999
≥50	4	2	2	
Sex				
Male	7	4	3	>0.9999
Female	6	3	3	
Tumor size				
<3 cm	3	1	2	0.5594
≥ 3 cm	10	6	4	
TNM stage				
I-II	8	6	2	0.1026
III-IV	5	1	4	

Note: miR-375: microRNA-375; HCC: hepatocellular carcinoma; TNM: tumor, node, metastases.

**Table 4:** The correlations of the HOXB3 level with HCC clinical features.

Clinical features	*n*	High HOXB3 (*n* = 7)	Low HOXB3 (*n* = 6)	*p* value
Age (years)				
<50	9	4	5	0.5594
≥50	4	3	1	
Sex				
Male	7	4	3	>0.9999
Female	6	3	3	
Tumor size				
<3 cm	3	2	1	>0.9999
≥3 cm	10	5	5	
TNM stage				
I-II	8	5	3	0.5921
III-IV	5	2	3	

Note: HOXB3: homeobox B3; HCC: hepatocellular carcinoma; TNM: tumor, node, metastases.

## Data Availability

The data used to support the findings of this study are included within the article.

## Supplementary Materials

Supplementary Materials Figure S1: exosomal miR-375 synergizes with sorafenib to inhibit the growth of HCC cells. (A) Detection of apoptotic ratio of Huh-7 cells by flow cytometry. (B) Detection of the proliferation ability of Huh-7 cells using EdU staining. ^#^*p* < 0.05 vs. Huh-7 cells treated with EV-miR-375 mimic+DMSO. All data were expressed as mean ± standard deviation of at least three independent experiments. Data analysis between the two groups was performed by unpaired *t*-test.
