# Supplementary material for: Extracellular Vesicle-Encapsulated MicroRNA-375 from Bone Marrow-Derived Mesenchymal Stem Cells Inhibits Hepatocellular Carcinoma Progression through Regulating HOXB3-Mediated Wnt/β-Catenin Pathway
Source: Anal Cell Pathol (Amst). 2022 Jan 27;2022:9302496. doi: 10.1155/2022/9302496 (PMC8813296; doi:10.1155/2022/9302496)
Supplement: Supplementary Materials — Figure S1: exosomal miR-375 synergizes with sorafenib to inhibit the growth of HCC cells. (A) Detection of apoptotic ratio of Huh-7 cells by flow cytometry. (B) Detection of the proliferation ability of Huh-7 cells using EdU staining. #p < 0.05 vs. Huh-7 cells treated with EV-miR-375 mimic+DMSO. All data were expressed as mean ± standard deviation of at least three independent experiments. Data analysis between the two groups was performed by unpaired t-test. [file 9302496.f1.docx]

**Figure S1**

**
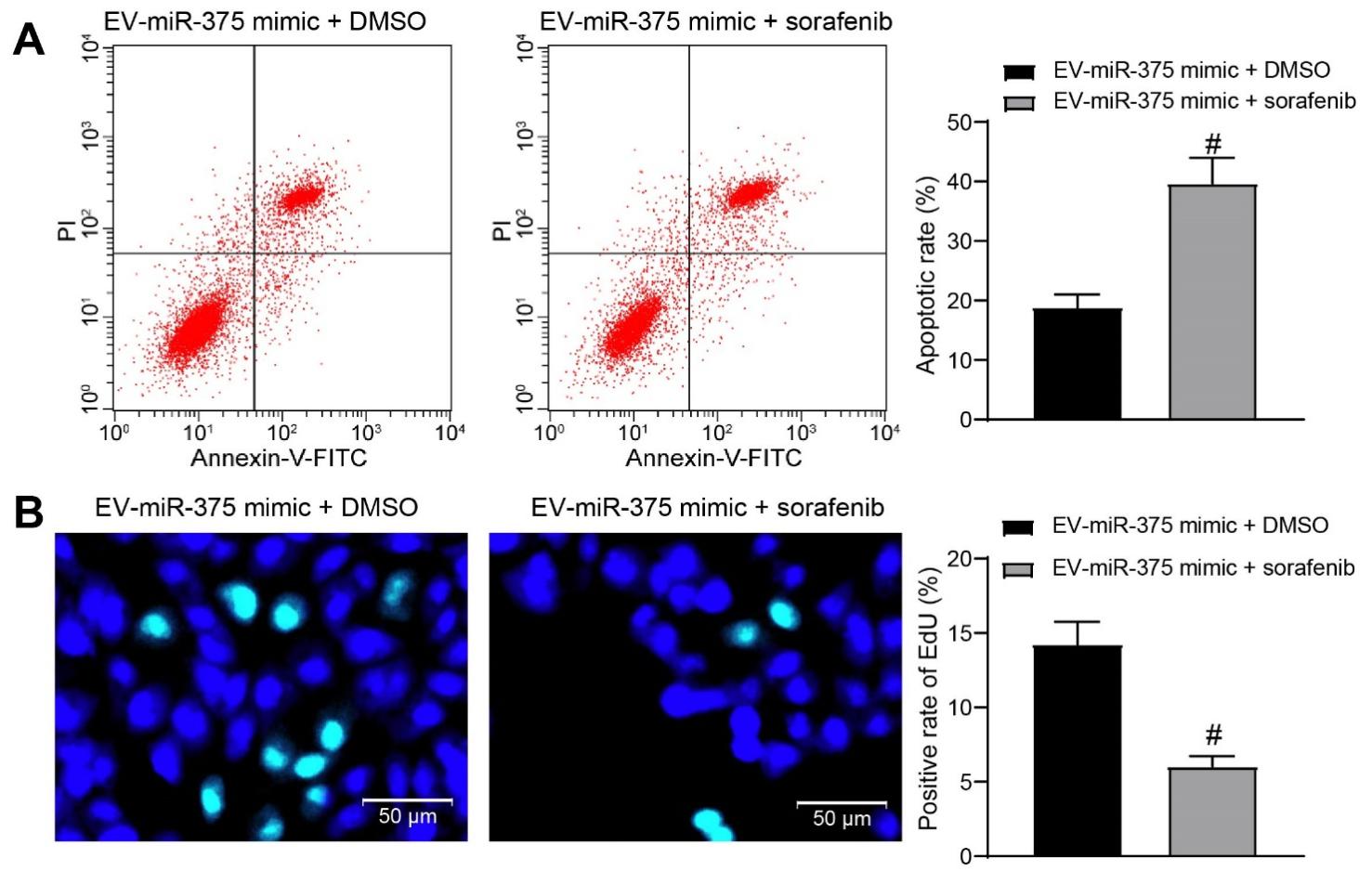
**

**Figure S1** Exosomal miR-375 synergizes with sorafenib to inhibit the growth of HCC cells. A. Detection of apoptotic ratio of Huh-7 cells by flow cytometry; B. Detection of the proliferation ability of Huh-7 cells using EdU staining. # *p* < 0.05 vs Huh-7 cells treated with EV-miR-375 mimic + DMSO. All data were expressed as mean ± standard deviation of at least three independent experiments. Data analysis between the two groups was performed by unpaired *t* test.
